# High-Proportion Blue Light Irradiation at the End-of-Production Stage Promotes the Biosynthesis and Recycling of Ascorbate in Lettuce

**DOI:** 10.3390/ijms242216524

**Published:** 2023-11-20

**Authors:** Chengbo Zhou, Zonggeng Li, Wenke Liu, Zhonghua Bian, Wei Lu, Bo Zhou, Sen Wang, Qingming Li, Qichang Yang

**Affiliations:** 1 Institute of Urban Agriculture, Chinese Academy of Agriculture Science, Chengdu 610213, China; zhouchengbo@caas.cn (C.Z.); lizonggeng@caas.cn (Z.L.); bianzhonghua@caas.cn (Z.B.); zhoubo02@caas.cn (B.Z.); wangsen@caas.cn (S.W.); liqingming@caas.cn (Q.L.); 2 Institute of Environment and Sustainable Development in Agriculture, Chinese Academy of Agricultural Sciences, Beijing 100081, China; 3 Key Lab of Energy Conservation and Waste Management of Agricultural Structures, Ministry of Agriculture and Rural Affairs, Beijing 100081, China; 4 College of Horticulture, Sichuan Agricultural University, Chengdu 611134, China; luwei-1213@163.com

**Keywords:** antioxidant, reactive oxygen species, light quality, plant factory, enzyme activity

## Abstract

Ascorbate (AsA), an essential antioxidant for both plants and the human body, plays a vital role in maintaining proper functionality. Light plays an important role in metabolism of AsA in horticultural plants. Our previous research has revealed that subjecting lettuce to high light irradiation (HLI) (500 μmol·m^−2^·s^−1^) at the end-of-production (EOP) stage effectively enhances AsA levels, while the optimal light quality for AsA accumulation is still unknown. In this study, four combinations of red (R) and blue (B) light spectra with the ratio of 1:1 (1R1B), 2:1 (2R1B), 3:1 (3R1B), and 4:1 (4R1B) were applied to investigate the biosynthesis and recycling of AsA in lettuce. The results demonstrated that the AsA/total-AsA content in lettuce leaves was notably augmented upon exposure to 1R1B and 2R1B. Interestingly, AsA levels across all treatments increased rapidly at the early stage (2–8 h) of irradiation, while they increased slowly at the late stage (8–16 h). The activity of L-galactono-1,4-lactone dehydrogenase was augmented under 1R1B treatment, which is pivotal to AsA production. Additionally, the activities of enzymes key to AsA cycling were enhanced by 1R1B and 2R1B treatments, including ascorbate peroxidase, dehydroascorbate reductase, and monodehydroascorbate reductase. Notably, hydrogen peroxide and malondialdehyde accumulation increased dramatically following 16 h of 1R1B and 2R1B treatments. In addition, although soluble sugar and starch contents were enhanced by EOP-HLI, this effect was comparatively subdued under the 1R1B treatment. Overall, these results indicated that AsA accumulation was improved by irradiation with a blue light proportion of over 50% in lettuce, aligning with the heightened activities of key enzymes responsible for AsA synthesis, as well as the accrual of hydrogen peroxide. The effective strategy holds the potential to enhance the nutritional quality of lettuce while bolstering its antioxidant defenses.

## 1. Introduction

Ascorbate (AsA) is a pivotal antioxidant group in leafy vegetables, functioning as a cofactor for several enzymes that contribute to various developmental processes [1,2]. However, leafy vegetables cultivated in protected environments often have lower levels of AsA due to reduced light irradiation in greenhouses or plant factories [1,3]. The absence of L-galactono-1,4-lactone dehydrogenase (GLDH), an enzyme pivotal to AsA production, necessitates human acquisition of AsA through daily diets for maintaining health [4]. Elevating AsA levels in vegetables not only extends shelf life but also enhances their nutritional value for human consumption [2,5]. Therefore, optimizing the light conditions for leafy vegetables carries immense significance in improving AsA levels and advancing protected cultivation.

Both biotic and abiotic stresses can rapidly increase reactive oxygen species (ROS) accumulation, disrupting the equilibrium between ROS generation and elimination in plants [6]. Importantly, excessive ROS accumulation can lead to membrane lipid peroxidation, nuclear degradation, and cell death in severe cases [7,8,9]. The induction of AsA accumulation serves as a mechanism to counteract ROS excess in plants [9]. As a nonenzymatic antioxidant, AsA directly reacts with ROS to reduce their levels [10]. In addition, the oxidation of AsA by ascorbate peroxidase (APX) facilitates H_2_O_2_ reduction and removal [11].

Light emerges as a critical important environmental factor regulating AsA levels in plants. High light irradiation (HLI, 500 μmol·m^−2^·s^−1^) increases AsA content in *Arabidopsis* [12], tomato [13,14], and lettuce [2,15], and improves the activities of enzymes key to AsA metabolism, including GLDH, dehydroascorbate reductase (DHAR), ascorbate peroxidase (APX), and monodehydroascorbate reductase (MDHAR), augmenting AsA biosynthesis and accumulation [15,16]. Notably, HLI leads to a marked rise in AsA content compared to darkness [13]. However, a nine-day treatment of light irradiation does not alter AsA levels in tomato fruits [13], while decreasing AsA content and inducing oxidative stress in lettuce [15]. Therefore, the concept of HLI at the end-of-production (EOP) stage introduced by Gómez and Jiménez (2020) has proven to be promising in enhancing lettuce’s nutritional quality in controlled environments [17]. The concept’s feasibility was further confirmed by increased AsA and carbohydrate contents in lettuce and prolonged shelf life under HLI before harvest [2].

Besides HLI, light quality influences nutritional outputs of horticultural plants [18,19]. Blue and red lights play crucial roles in regulating plant morphogenesis, carbohydrate accumulation, and secondary metabolism through phytochromes, phototropins, and cryptochromes [20,21,22]. The integration of red and blue lights has become common in plant factories because of its capacity to augment yield and produce horticultural products with high quality [23,24]. Studies have explored the impacts of red-to-blue (R-to-B) ratios on AsA accumulation in different plants and indicated that a high red light proportion promotes the AsA level in broccoli [25] and celery [26], while a high blue light proportion increases the AsA content in lettuce [27]. However, the lights were used during post-harvest or as supplemental lighting. In addition, although our previous study demonstrated that the R-to-B ratio of 1:3 with light intensity of 200 μmol·m^−2^·s^−1^ enhanced AsA content in lettuce as compared with 3:1 and 1:1 [28], the optimal light quality for AsA accumulation was not determined, and a more practical strategy should be explored.

The high energy cost in plant factory production is an urgent problem that needs to be addressed. EOP-HLI holds the potential to save energy and improve nutritional quality of products at the same time. We have previously shown that EOP-HLI increases AsA levels in lettuce leaves [16]. To further optimize the light condition and improve AsA accumulation, it is necessary to investigate various R-to-B ratios under the condition of EOP-HLI. Overall, this study aims to obtain an effective light condition that can be used for production of horticultural plants in a controlled environment or plant factory.

## 2. Results

### 2.1. Ascorbate Pool

**Figure 1:**
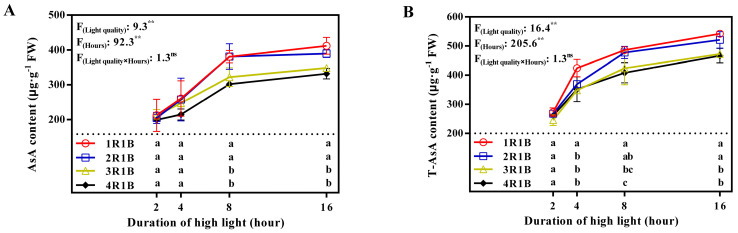
Temporal trends of AsA (**A**) and total−AsA (**B**) levels in lettuce leaves under EOP−HLI with varying R−to−B ratios. All data are the means ± SD of four repeats. Different letters at given time points denote *p* < 0.05. Inset provides the F values and significance derived from two−way ANOVA of light quality, duration, and their interactions. ^ns^ and ** indicate insignificant and *p* < 0.01, respectively.

1R1B and 2R1B treatments were more conducive to AsA accumulation in lettuce compared with 3R1B and 4R1B treatments. AsA and total-AsA levels were augmented significantly in lettuce leaves following EOP-HLI for 16 h, especially during 2–8 h (Figure 1). Over this period, AsA content increased by 51.5%–86.1% (1R1B: 79.3%; 2R1B: 86.1%; 3R1B: 51.9%; 4R1B: 51.5%), and total-AsA increased by 56.3%–78.6% (1R1B: 77.9%; 2R1B: 78.6%; 3R1B: 72.6%; 4R1B: 56.3%). AsA and total-AsA levels were moderately steady after the first 8 h of EOP-HLI. After 16 h of EOP-HLI, AsA contents were 412.0 μg·g^−1^ FW in 1R1B and 389.8 μg·g^−1^ FW in 2R1B, which were significantly elevated compared to 348.2 μg·g^−1^ FW in 3R1B and 331.6 μg·g^−1^ FW in 4R1B. Similarly, total-AsA content was also significantly augmented in 1R1B (542.5 μg·g^−1^ FW) and 2R1B (521.0 μg·g^−1^ FW) treatments compared to 3R1B (472.7 μg·g^−1^ FW) and 4R1B (466.9 μg·g^−1^ FW) treatments at 16 h.

### 2.2. Enzyme Activity

**Figure 2:**
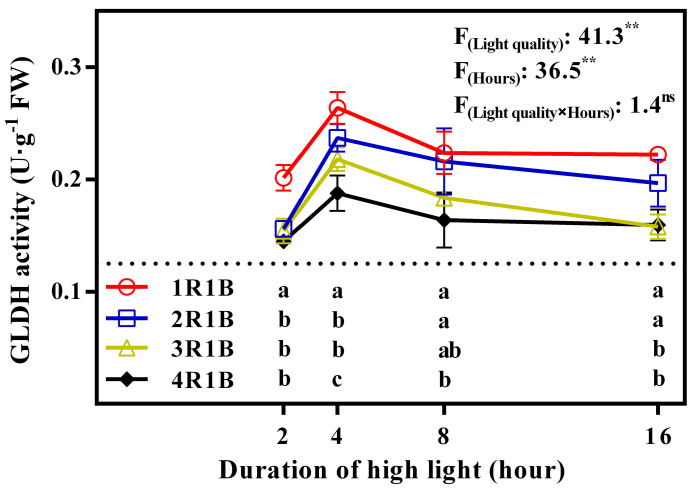
Temporal changes in GLDH activity in lettuce leaves under EOP−HLI with varying R−to−B ratios. All data are the means ± SD of four repeats. Different letters at given time points denote *p* < 0.05. Inset provides the F values and significance derived from two−way ANOVA of light quality, duration, and their interactions. ^ns^ and ** indicate insignificant and *p* < 0.01, respectively.

**Figure 3:**
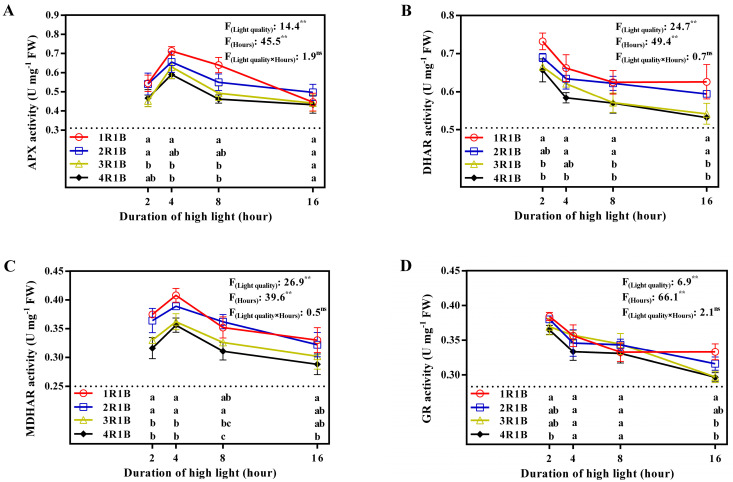
Temporal changes in APX (**A**), DHAR (**B**), MDHAR (**C**), and GR (**D**) activities in lettuce leaves under EOP−HLI with varying R−to−B ratios. All data are the means ± SD of four repeats. Different letters at given time points denote *p* < 0.05. Inset provides the F values and significance derived from two−way ANOVA of light quality, duration, and their interactions. ^ns^ and ** indicate insignificant and *p* < 0.01, respectively.

The GLDH activity in lettuce was the highest at 4 h under 1R1B treatment, surpassing activity under the 2R1B, 3R1B and 4R1B treatments by 11.3%, 20.9%, and 40.5%, respectively. GLDH activity increased at 4 h and then reduced (Figure 2). APX activity reached its peak at 4 h under the 1R1B treatment, which was 8.9%, 13.3%, and 20.8% higher than that under the 2R1B, 3R1B, and 4R1B treatments, respectively. Similar to GLDH, APX activity of lettuce peaked from 2 h to 4 h and then declined gradually (Figure 3A). DHAR activity of lettuce decreased with increased length of time under EOP-HLI. In general, higher DHAR activity of lettuce was observed under high-proportion blue light, especially at 8 h and 16 h (Figure 3B). Under 2 or 4 h of EOP-HLI, MDHAR activity was significantly elevated under 1R1B and 2R1B compared to 3R1B and 4R1B (Figure 3C). While no notable difference in GR activity was detected in the early stage of EOP-HLI, GR activity in the later stage (16 h) was significantly higher under 1R1B than under 3R1B and 4R1B (Figure 3D).

### 2.3. Soluble Sugar and Starch Levels

**Figure 4:**
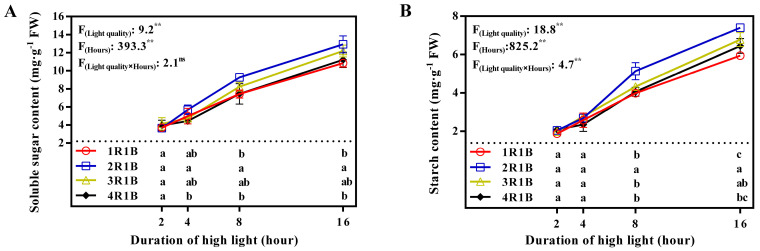
Temporal trends of soluble sugar (**A**) and starch (**B**) levels in lettuce leaves under EOP−HLI with varying R−to−B ratios. All data are the means ± SD of four repeats. Different letters at given time points denote *p* < 0.05. Inset provides the F values and significance derived from two−way ANOVA of light quality, duration, and their interactions. ^ns^ and ** indicate insignificant and *p* < 0.01, respectively.

EOP-HLI significantly elevated the soluble sugar levels in lettuce leaves, with a progressive rise in correspondence with extended irradiation duration. Under 16 h of HLI, its accumulation reached a maximum of 10.8, 12.9, 12.2, and 11.2 mg·g^−1^ FW in 1R1B, 2R1B, 3R1B, and 4R1B, respectively. Notably, no notable difference was observed at the 2 h mark. However, after the initial 4 h of EOP-HLI, the 2R1B treatment exhibited the highest soluble sugar content (Figure 4A). Comparable patterns were observed for starch content at the 2 h and 4 h intervals across various treatments. In the late stages (8 and 16 h) of EOP-HLI, the starch level was dramatically higher in 2R1B than in 1R1B and 4R1B treatments (Figure 4B).

### 2.4. O_2_^−^, H_2_O_2_, and MDA Contents

**Figure 5:**
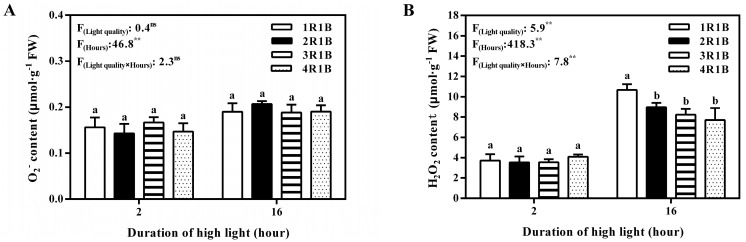
Temporal changes in O_2_^−^ (**A**) and H_2_O_2_ (**B**) levels in lettuce leaves under EOP−HLI with varying R−to−B ratios. All data are the means ± SD of four repeats. Different letters at given time points denote *p* < 0.05. Inset provides the F values and significance derived from two−way analysis of variance of light quality, duration, and their interactions. ^ns^ and ** indicate insignificant and *p* < 0.01, respectively.

**Figure 6:**
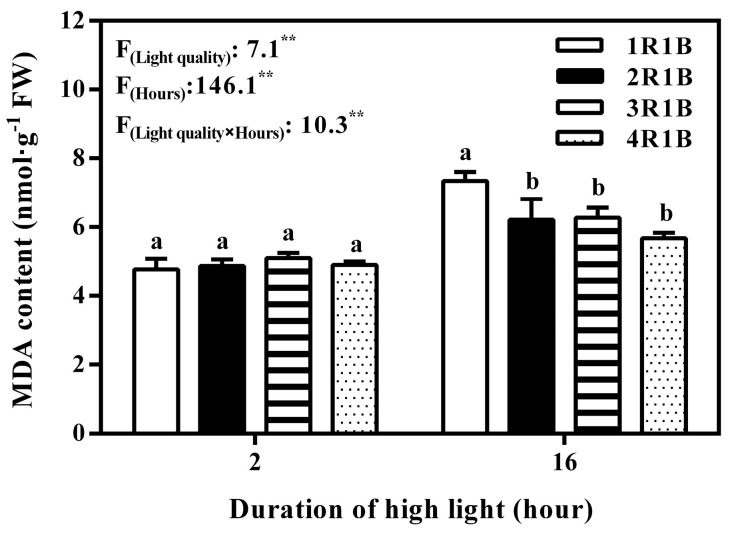
Temporal changes in MDA level in lettuce leaves under EOP−HLI with varying R−to−B ratios. All data are the means ± SD of four repeats. Different letters at given time points denote *p* < 0.05. Inset provides the F values and significance derived from two−way ANOVA of light quality, duration, and their interactions. ** indicate *p* < 0.01.

Compared with 2 h of EOP-HLI, O_2_^−^ level in lettuce leaves was increased at 16 h (Figure 5A). Similar results were found in H_2_O_2_ content at 2 h, but the content of H_2_O_2_ was the highest (10.7 μmol·g^−1^ FW) at 16 h under 1R1B treatment. In addition, compared with O_2_^−^, the content of H_2_O_2_ was increased more obviously after EOPHLI for 16 h (Figure 5B). No obvious difference in MDA level was noticed among treatments at 2 h. At 2 h, MDA content was higher than at 16 h across all treatments. The MDA level in lettuce leaves was significantly augmented under the 1R1B treatment compared to the other treatments after 16 h of HLI (Figure 6).

## 3. Discussion

Light serves as a pivotal environmental determinant influencing the process of plant growth. The interplay of light intensity and its compositions intricately governs plant growth and developmental processes. Many studies have shown that light quality could affect plant photosynthesis and quality, affect plant growth by regulating the distribution of dry matter, and enhance stress tolerance [29,30,31]. Similarly, our previous research found that the highest AsA and total-AsA levels in lettuce under EOP-HLI were 371 and 503 μg·g^−1^ FW, respectively [16]. In this study, R-to-B ratios in EOP-HLI were optimized to further improve the enhancing effects of EOP-HLI on the lettuce AsA levels. The highest AsA and total-AsA levels were 412.0 and 542.5 μg·g^−1^ FW, respectively, under EOP-HLI with 1R1B (high-proportion blue light). Therefore, AsA and total-AsA were further enhanced by 11.1% and 7.9% as compared with our previous research. This finding is consistent with previous results in which the AsA level in lettuce leaves was increased by continuous light with a high ratio of blue light [28]. In addition, long-term HLI and a high proportion of blue light are unfavorable for growth of plants [32,33]. Our study revealed that distinct red and blue light combinations evince no dramatic impacts on the growth of hydroponic lettuce (Table A1), indicating that 16 h of EOP-HLI was not sufficient to affect the growth of lettuce. This result was also confirmed in our previous research [16]. Overall, EOP-HLI with 1R1B was a practical strategy to cultivate lettuce with high AsA content without sacrificing the yield.

### 3.1. EOP-HLI with a High Blue Light Proportion Increased AsA Accumulation by Promoting Its Biosynthesis and Recycling

High-proportion blue light treatment (1R1B and 2R1B) significantly enhanced AsA and total-AsA levels in lettuce, especially via EOP-HLI for 8 and 16 h (Figure 1). To determine the changes during AsA biosynthesis and the regeneration process, the activities of key enzymes were determined. GLDH catalyzes the final step of AsA biosynthesis and its activity is positively correlated with AsA content across diverse tissues in a species-specific manner [34,35]. Light plays important roles in the expression of GLDH [36]. In this study, GLDH activity increased under high-proportion blue light treatment (Figure 2), indicating that blue light might increase the activity of GLDH. Interestingly, high-proportion red light under continuous light and a high nitrogen level increased the activity of GLDH [37]. This may be because enzyme activity was comprehensively regulated by light quality and nitrogen levels. APX, MDHAR, DHAR, and GR are pivotal in AsA recycling because they mediate AsA degradation and regeneration (MDHA-AsA and DHA-AsA recycling) [38,39,40]. Our study revealed that the activities of APX, MDHAR, and DHAR, which promote the recycling of the AsA pool, were higher under EOP-HLI with high-proportion blue light (Figure 3A–C). Furthermore, higher GR activity was observed under high-proportion blue light at 16 h (Figure 3D), further indicating the synergistic regulatory effect of DHAR and GR in DHA-AsA cycling. Similar results were observed in *Arabidopsis* under high light levels [12]. The accelerated recycling of the AsA pool can not only improve AsA levels but also remove ROS in lettuce [9]. Therefore, lettuce treated with EOP-HLI with a high blue light proportion has the potential to prolong the shelf life. Similar to GLDH, the activities of APX, MDHAR, DHAR, and GR were reported to be higher under high-proportion red light with continuous light and a high nitrogen level [37]. However, He et al. [41] reported that the activities of the aforementioned enzymes increased under high-proportion blue light. It was speculated that nitrogen contributes more to GLDH activity compared with light spectra.

### 3.2. AsA Level Was Influenced by Carbohydrate Accumulation within the Range of Blue Light Accounting for No More than 33%

It is well-established that high light intensity and quality significantly improve the carbohydrate level in plants [2,42]. The conversion of soluble sugar and starch to glucose and fructose can provide substrates and energy for AsA biosynthesis in the D-mannose/L-galactose pathway [43,44]. As a downstream metabolite of glucose, the biosynthesis of AsA would likely be affected by increased carbohydrates [3]. However, studies also suggested that light-induced AsA accumulation is independent of carbohydrate availability in *Arabidopsis* and tomato [3,44]. This is probably because carbohydrate availability might not serve as a limiting factor for AsA accumulation when carbohydrates accumulate as substrates to a certain extent. Therefore, we investigated the carbohydrate accumulation in different treatments.

The proportion of blue light in 1R1B, 2R1B, 3R1B, and 4R1B treatments was 50%, 33%, 25%, and 20%, respectively. As the proportion of blue light increased from 20% to 33%, the content of carbohydrates gradually increased at 8 or 16 h (Figure 4). Similar results were observed in the AsA levels (Figure 1). As the proportion of blue light increased to 50% (1R1B), the carbohydrate content in lettuce leaves was significantly lower at 8 or 16 h as compared with that in other treatments, while the AsA level was still the highest in the 1R1B treatment. In terms of the light-regulated carbohydrate content, several studies also proved that high-proportion red light improved contents of carbohydrates such as glucose, fructose, sucrose, and starch [45,46]. It was speculated that the decrease in the proportion of red light limited the accumulation of carbohydrates to a certain extent, as red light is considered to be a more effective wavelength for photosynthesis [24]. On the other hand, the results proved the hypothesis mentioned above that carbohydrate availability might not serve as a limiting factor for AsA accumulation when carbohydrates accumulate to a certain extent. Similarly, it was also observed that the carbohydrate content was not a determinant in the regulation of AsA content in *Arabidopsis* [47] and tomato [3]. Overall, it can be speculated that when the proportion of blue light exceeded 33%, AsA accumulation was regulated by enzyme activities in its metabolism rather than by carbohydrate metabolism.

### 3.3. The AsA Accumulation in Lettuce Induced by EOP-HLI with a High Blue Light Proportion Was Correlated with ROS

Previous studies pointed out that high-energy blue light increased osmotic regulators to help stabilize proteins and alleviate peroxide stress [48,49]. Furthermore, AsA regeneration is an important system to maintain the metabolic balance of ROS in plants [39,50]. AsA is a powerful antioxidant for scavenging ROS because it can donate electrons in a series of enzymatic and non-enzymatic reactions [51]. In this study, higher AsA levels were observed under EOP-HLI with a high blue light proportion (1R1B and 2R1B), and this elevation is pivotal for ROS scavenging as a stress response. Excess light energy under high-proportion blue light led to the production of ROS in lettuce leaves because the H_2_O_2_ and MDA contents increased after 16 h of EOP-HLI (Figure 5 and Figure 6). Our results were consistent with previous reports that blue light induced enzymatic conversion of molecular oxygen to ROS [52,53]. The present study indicated that the AsA accumulation in lettuce induced by high-proportion blue light was correlated with ROS. This could be partly supported by the evidence that higher activities of APX, DHAR, and MDHAR were all detected under high-proportion blue light treatment (Figure 3) because the work of these enzymes is accompanied by the decomposition of ROS [54]. Therefore, blue light can be used to improve the quality of cultivated vegetables and, as suggested by El-Esawi et al. [55], can prime plants against the deleterious effects of environmental stresses.

**Figure 7:**
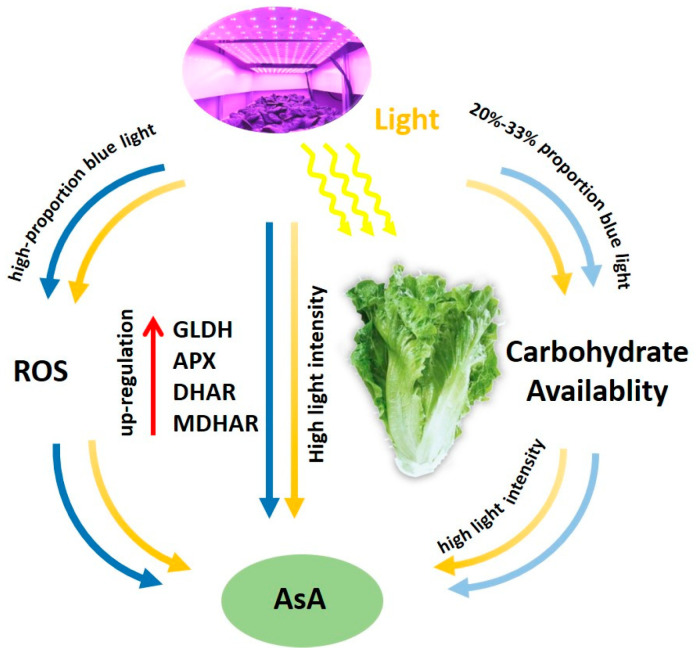
Simplified illustration of AsA regulation processes under EOP-HLI with different red and blue combinations in lettuce.

In general, AsA recycling in plants is mainly mediated by MDHAR. However, under stress conditions, the oxidation rate of AsA exceeds the MDHAR activity, which increases the possibility of synergistic regulation between AsA and glutathione [12,56,57]. Indeed, the oxidation status of glutathione is critical for AsA recycling as glutathione provides electrons for the reduction of DHA [1]. Moreover, glutathione also participates in many metabolic regulation and antioxidant processes and is vital to the ROS defense system [58]. This study revealed that the H_2_O_2_ level was increased under EOP-HLI with a high blue light proportion at 16 h. Then, the activities of GR and DHAR increased (Figure 3), suggesting that the AsA-GSH cycle mediated by GR and DHAR is crucial under high proportions of blue light. Hence, it was speculated that high-proportion blue light under EOP-HLI increased ROS accumulation in lettuce, and this signal had a positive effect on the recycling of AsA to remove excess ROS (Figure 7).

## 4. Materials and Methods

### 4.1. Plants and Light Treatments

Lettuce (*Lactuca sativa* L. cv. ‘Tiberius’) seeds were meticulously placed in germination trays, each containing moist sponge cube blocks of 2.5 × 2.5 × 2.5 cm. A singular seed was sown within each cube. The cubes were placed in a chamber in a plant factory at 22 ± 1 °C with 72 ± 2% relative humidity in the dark for the initial two days and thereafter under white LED (200 μmol·m^−2^·s^−1^) irradiation with a 16/8hours light/dark cycle. The white LED light comprised 15.8% blue (400–500 nm), 22.0% green (500–600 nm), 50.3% red (600–700 nm), and 11.8% far-red (700–800 nm) lights, as described previously [31].

Upon the second leaf unfolding (15 days after sowing), seedlings with similar morphology were transplanted to a designed recirculating hydroponic system supplied with LED panels emitting red or blue lights and cultivated with 27 plants/m^2^ in the plant factory with day/night temperature of 24 ± 1/22 ± 1 °C, relative humidity of 65 ± 5%, 423 ± 2 μmol mol^−1^ CO_2_, and a photoperiod having a 16 h light period (6:00–22:00) and an 8 h dark period (22:00–6:00). A modified Hoagland nutrient solution (1.63 mS·cm^−1^, pH 5.7) of 4 mM Ca(NO_3_)_2_·4H_2_O, 6 mM KNO_3_, 1 mM NH_4_H_2_PO_4_, 2 mM MgSO_4_·7H_2_O, 71 μM Fe-EDTA-Na_2_, 46 μM H_3_BO_3_, 9.6 μM MnSO_4_·4H_2_O, 0.8 μM CuSO_4_·5H_2_O, and 0.07 μM (NH_4_)_6_Mo_7_O_24_·4H_2_O was prepared using purchased deionized water (EC: 0 mS·cm^−1^, pH: 5.98), and applied to lettuce cultivation via daily circulation in the system and refreshed every seven days during the experiment.

**Figure 8:**
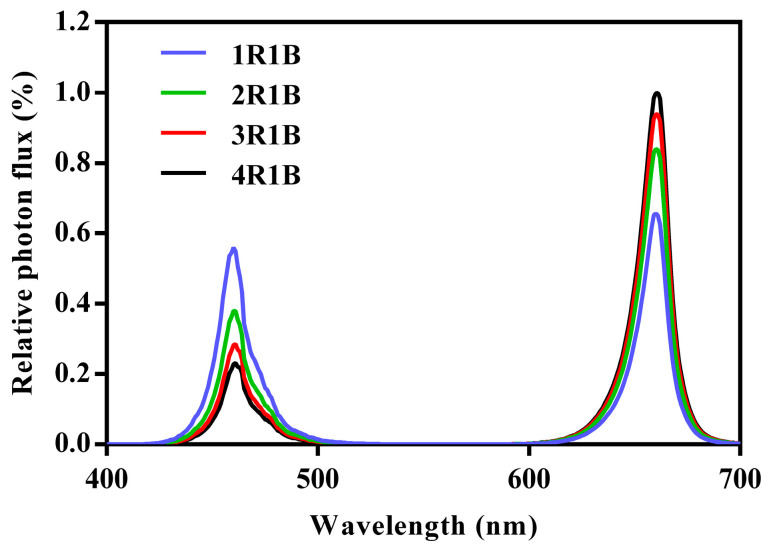
Spectra of the employed red and blue LED panels.

**Figure 9:**
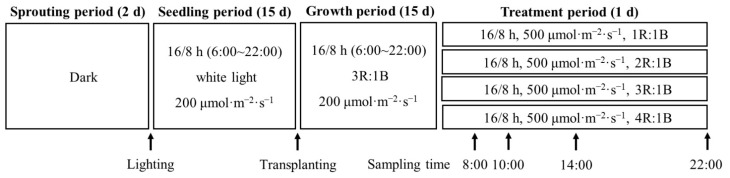
Schematic diagram delineating various growth stages, arrangements, and designated sampling instances for the end-of-production high light experiment featuring various R-to-B ratios.

In the growth room, two 180 cm long, 70 cm wide, and 200 cm high cultivation frames were securely positioned, each equally subdivided into six cultivation units in three layers. The study utilized the top four units. To prevent light contamination, each unit was enveloped in opaque plastic films. A 50 × 50 cm LED light panel (Wuxi Huazhaohong Optoelectronic Technology Co., Ltd., Wuxi, China) that emitted lights with peak wavelengths of 662 nm (red) and 460 nm (blue) was positioned 30 cm above the hydroponic pots. Light intensity was checked using spectroradiometers (Avaspec-ULS2048, Avantes, Apeldoorn, The Netherlands) installed 10 cm above each hydroponic pot and maintained at 200 μmol·m^−2^·s^−1^ with an R-to-B ratio of 3:1, a configuration deemed suitable for optimal lettuce growth, as supported by prior research [18]. After cultivation for 15 days, the plants were assigned by chance to four groups with 39 plants each. Commencing at 6:00 a.m. on the 16th day, the light intensity was elevated to 500 μmol·m^−2^·s^−1^ with an R-to-B ratio of 1:1 (1R1B), 2:1 (2R1B), 3:1 (3R1B), and 4:1 (4R1B) (Figure 8 and Figure 9). Subsequent to specific periods of irradiation (2 h at 8:00, 4 h at 10:00, 8 h at 14:00, and 16 h at 22:00), four to six fully unfolded leaves without petioles were picked from each of four randomly selected plants from each group (Figure 9) to form a single biological replicate. They were snap frozen and preserved at −80 °C before subsequent assays.

### 4.2. Destructive Measurements

Destructive measurements were carried out one day after the treatment. To minimize shading effects between larger plants, plants were harvested in an earlier growth period than those typically used in commercial cultures. Four plants were randomly chosen, and their stems, leaves, and roots were separately collected to assess leaf area using a leaf area meter (LI-3100C, Li-Cor Biosciences, Lincoln, NE, USA), and to weigh fresh leaves, shoots, and roots. Subsequently, the leaves, roots, and stems were incubated for 48 h at 80 °C to assess dry matter.

### 4.3. Soluble Sugar and Starch Determination

Leaf soluble sugar was extracted by boiling 0.1 g of fresh leaves in 1 mL of deionized water after homogenization. After spinning for 10 min at 8000× *g*, OD_620 nm_ of the supernatant was measured to calculate soluble sugar content.

Leaf starch was gelatinized by boiling 0.1 g of frozen leaves in 100 μL of distilled water for 30 min after homogenization. At 23 °C, the product was supplied with 100 μL of 9.2 M perchloric acid and 200 μL of distilled water, stirred for 10 min, and spun for 6 min (8000× *g*) at room temperature. The precipitates were washed with 100 μL of 4.6 M perchloric acid and 300 μL of distilled water under continuous stirring for 10 min and spun. After three washes, the supernatants were combined, and OD_620 nm_ was measured to assess starch content.

### 4.4. Determination of AsA and Total-AsA

AsA and total-AsA levels in lettuce were quantified following a modified protocol [59,60]. Crude AsA was extracted from 0.1 g fresh leaves in 1 mL of cold solution containing 0.5 mM EDTA, 1.5% metaphosphoric acid, and 4% acetic acid and spun at 4 °C for 10 min at 15,000× *g*. A quantity of 50 μL of the extraction was supplied with 50 μL of 0.4 M sulfuric acid, 190 μL of 200 mM Tris buffer, and 10 μL of ultrapure water. After incubation at 23 °C for 30 min, AsA was purified by being passed through 0.22 μm polytetrafluoroethylene filters, and separated on an Acquity UPLC system supplied with an HSS T3 column (2.1 × 100 mm, 1.8 mm, Waters, Milford, MA, USA) at 0.25 mL·min^−1^ using 0.1% (*v*/*v*) formic acid as the mobile phase. OD_245 nm_ was measured to determine AsA levels based on a L-ascorbic acid standards. Total-AsA content was determined similarly by replacing ultrapure water with 10 μL of 750 mM dithiothreitol.

### 4.5. Enzyme Extraction and Activity Measurement

To assess GLDH activity, crude enzymes were prepared by grinding 0.3 g fresh leaves in 2 mL of 100 mM PBS (pH 7.4) with 0.4 M sucrose, 10% (*v*/*v*) glycerol, 1 mM EDTA, 0.3% (*v*/*v*) mercaptoethanol, and 1% (*w*/*v*) PVP. Afterward, 100 μL of the crude enzyme was combined with 1 mL of 50 mM PBS (pH 7.8) with 1.05 mg·mL^−1^ Cyt c and 5.6 mM L-GAL. The reaction was triggered by addition of L-GAL and monitored by measuring OD_550 nm_. The GLDH activity unit was specified as the enzyme amount for reducing 1 μmol of Cyt c or oxidizing 1 mmol of L-GAL per min. APX activity was determined according to the method of Zha et al. (2019) [4]. To assess DHAR, MDHAR, and GR activities, extraction was performed using the method of Zhou et al. (2021) [16] and the enzymatic activity was carried out following the method of Ma and Cheng [61].

### 4.6. Measurement of H_2_O_2_ and O_2_^−^ Contents

The H_2_O_2_ level was quantified using a kit following the protocol (Comin Biotechnology, Suzhou, China). Fresh leaves (0.1 g) were extracted with 1 mL of precooled 100% acetone and spun (8000× *g*, 4 °C) for 10 min. OD_415 nm_ was determined to assess the H_2_O_2_ level. The O_2_^−^ level was assessed as described previously [18].

### 4.7. Measurement of Malondialdehyde Levels

Malondialdehyde (MDA) levels were assessed as described previously [62]. A quantity of 0.1 g of fresh leaves was extracted in 1 mL of cold 10% trichloroacetic acid and spun (8000× *g*, 4 °C) for 10 min. About 500 μL of the supernatant was kept at 100 °C for 30 min. After cooling to 23 °C and spinning (10,000× *g*) for 10 min, OD_450 nm_, OD_532 nm_, and OD_600 nm_ were measured to assess the MDA level.

### 4.8. Statistical Analysis

The effects of two variables, namely ‘light quality’ and ‘hours’, along with their interaction, were determined using a two-way analysis of variance. Statistically significant disparities among various treatments were assessed using Duncan’s multiple range test with 95% confidence in SPSS 23 (SPSS, Inc., Chicago, IL, USA).

## 5. Conclusions

Overall, on the basis of EOP-HLI, a high blue light proportion exhibited no discernible impact on lettuce growth. On the one hand, EOP high-proportion blue light irradiation promoted AsA accumulation in lettuce, which was correlated with increased activities of key enzymes in AsA biosynthesis and recycling. On the other hand, EOP-HLI with high-proportion blue light irradiation for 16 h increased H_2_O_2_ content in lettuce leaves to promote the circulating metabolism of AsA, and thus remove excessive ROS. EOP-HLI with a lower R-to-B ratio increased carbohydrate accumulation in lettuce, but did not impact AsA accumulation. Therefore, EOP-HLI could improve AsA level in lettuce, and a high blue light proportion (1R1B and 2R1B) had greater potential for increasing AsA levels.

## Data Availability

The datasets generated for this study are available on request to the corresponding author.

## Appendix A

**Table A1:** The biomass of lettuce under EOP-HLI with various R-to-B ratios.

Treatments	Leaf Area (cm^2^)	Shoot Fresh Weight (g)	Shoot Dry Weight (g)	Root Fresh Weight (g)	Root Dry Weight (g)
1R1B	446.03 ± 37.48 ns	27.62 ± 0.69 ns	1.43 ± 0.04 ns	3.09 ± 0.39 ns	0.173 ± 0.01 ns
2R1B	468.33 ± 5.01 ns	28.24 ± 1.36 ns	1.43 ± 0.13 ns	3.55 ± 0.48 ns	0.212 ± 0.02 ns
3R1B	464.90 ± 45.65 ns	31.75 ± 1.67 ns	1.52 ± 0.09 ns	3.56 ± 0.06 ns	0.206 ± 0.02 ns
4R1B	475.03 ± 59.85 ns	31.54 ± 4.76 ns	1.61 ± 0.08 ns	3.57 ± 0.38 ns	0.220 ± 0.06 ns

Notes: Data are means ± SD of four replicates. Different letters within rows indicate *p* ≤ 0.05 in Duncan’s multiple range test. ns indicate insignificant.
